# Metformin Induces Cell Cycle Arrest, Reduced Proliferation, Wound Healing Impairment *In Vivo* and Is Associated to Clinical Outcomes in Diabetic Foot Ulcer Patients

**DOI:** 10.1371/journal.pone.0150900

**Published:** 2016-03-10

**Authors:** Fatima Ochoa-Gonzalez, Alberto R. Cervantes-Villagrana, Julio C. Fernandez-Ruiz, Hilda S. Nava-Ramirez, Adriana C. Hernandez-Correa, Jose A. Enciso-Moreno, Julio E. Castañeda-Delgado

**Affiliations:** 1 Unidad de Investigación Biomédica de Zacatecas, Instituto Mexicano del Seguro Social (IMSS), Zacatecas, Zac., México; 2 Unidad Académica de Ciencias Químicas, Área de Ciencias de la Salud, Universidad Autónoma de Zacatecas, Zacatecas, México; 3 Unidad de Investigación Biomédica de Zacatecas, Instituto Mexicano del Seguro Social (IMSS), Zacatecas, Zac., México; 4 Área de ciencias de la salud, Universidad Autónoma de Zacatecas. Zacatecas, Zac., México; Harvard Medical School, UNITED STATES

## Abstract

**Background:**

Several epidemiological studies in diabetic patients have demonstrated a protective effect of metformin to the development of several types of cancer. The underlying mechanisms of such phenomenon is related to the effect of metformin on cell proliferation among which, mTOR, AMPK and other targets have been identified. However, little is known about the role that metformin treatment have on other cell types such as keratinocytes and whether exposure to metformin of these cells might have serious repercussions in wound healing delay and in the development of complications in diabetic patients with foot ulcers or in their exacerbation.

**Material and Methods:**

HaCaT Cells were exposed to various concentrations of metformin and cell viability was evaluated by a Resazurin assay; Proliferation was also evaluated with a colony formation assay and with CFSE dilution assay by flow cytometry. Cell cycle was also evaluated by flow cytometry by PI staining. An animal model of wound healing was used to evaluate the effect of metformin in wound closure. Also, an analysis of patients receiving metformin treatment was performed to determine the effect of metformin treatment on the outcome and wound area. Statistical analysis was performed on SPSS v. 18 and GraphPad software v.5.

**Results:**

Metformin treatment significantly reduces cell proliferation; colony formation and alterations of the cell cycle are observed also in the metformin treated cells, particularly in the S phase. There is a significant increase in the area of the wound of the metformin treated animals at different time points (P<0.05). There is also a significant increase in the size and wound area of the patients with diabetic foot ulcers at the time of hospitalization. A protective effect of metformin was observed for amputation, probably associated with the anti inflammatory effects reported of metformin.

**Conclusions:**

Metformin treatment reduces cell proliferation and reduces wound healing in an animal model and affects clinical outcomes in diabetic foot ulcer patients. Chronic use of this drug should be further investigated to provide evidence of their security in association with DFU.

## Introduction

Diabetes is a metabolic disease characterized by hyperglycemia and alterations in metabolism of lipids, carbohydrates and insulin resistance and is one of the leading causes of death worldwide for non-communicable diseases [1]. In 2013 an estimated of about 382 million people lived with DM worldwide and among them about 90% is type II diabetes mellitus (DM2). Estimations for 2035 with current trends indicate that there will be 592 million cases of DM2 worldwide [2].

It is known that DM2 is associated with higher risk of developing cardiovascular complications, vascular insufficiency, renal damage, retinopathy and diabetic foot ulcers (DFU). All of these complications have been reported to be associated with reduced quality of life of diabetic patients [3] and increased costs for healthcare systems [4–7].

Among complications of diabetics that impact heavily in costs and quality of life of the patients (although not the only one) are diabetic foot ulcers. It is known that diabetic foot ulcers remain the major cause of non-traumatic amputation worldwide [8, 9]. Among diabetics there is a 25% life-time chance of developing a DFU and it has been estimated that this complication has prevalence in diabetics ranging from 4.4 to 10.5% in different populations [10].

Several risk factors have been associated with increased risk of amputation in patients with diabetes such as neuropathy, ulcer severity (hazard ratio (HR): 7.99; confidence interval (CI): 3.12 to 20.47), peripheral artery disease (HR: 2.64; CI: 1.52 to 4.59) [11], infection and diabetes control (measured as Hb1ac%) [12, 13]. Also the role of chronicity in DFU is not well explored but might be associated with increased incidence of amputations in diabetic patients and it has been described that ulcer size and depth is a risk factor for amputation [12].

On the other hand, there has been some attention on several drugs used for diabetes treatment, particularly metformin, given that a reduction in the incidence of several non-communicable diseases in diabetic subjects treated with it. Since 2008 there is clear data showing a reduction in the incidence of several types of cancer in patients with diabetes treated with metformin [14–17]. This reduction in incidence was explored at the molecular level describing the mechanisms underlying such effect. It was described in several publications that the mechanism by which metformin inhibited proliferation of cancer cells both *in vitro* and *in vivo* in several models of cancer was mediated by inhibition of the AMPK pathway, mTOR inhibition and CyclinD1 inhibition of the cell cycle [18–21], explaining the reduced incidence of cancer in this patients.

Taken together, it is plausible that given the role of metformin in cell proliferation and the fact that wound closure and healing depend on the cell proliferation of the keratinocytes and wound closure that has been described for other cell types, metformin might also affect proliferation of cell types important for the DFU healing and therefore it might be important as a determinant of amputation in diabetic patients with ulcers. Therefore the aim of the present study was to determine in several settings the role of metformin in cell proliferation, wound healing and outcomes (such as ulcer size and amputation) in patients with diabetic foot ulcers.

## Material and Methods

### Drugs, chemicals and reagents

Metformin hydrochloride, Resazurin sodium salt, propidium iodide (PI), Trypan Blue (cell culture tested Sigma-Aldrich, St. Louis, MO, USA), Dulbecco´s modified Eagle medium (DMEM), Tripsin, L-glutamine, penicillin/streptomycin (Corning-Mediatech, Manasas USA), Fetal Bovine serum (FBS, Thermo-HyClone, Utah, USA), CFSE cell proliferation kit from (Invitrogen, CA, USA).Prednisolone (Sophia, Guadalajara Jalisco, México), Sevoflurane anesthetic (Abbott, Quebec, Canada),

### Cell culture and viability assays

Human Keratonocyte cell line HaCaT [22] cells were cultured in Dulbecco´s modified Eagle medium (DMEM,) supplemented with penicillin (100 units/ml) streptomycin (100 μg/ml) and 10% v/v heat inactivated fetal bovine serum (FBS) and 2 mM of L-glutamine. Cells were incubated at 37°C in a humidified atmosphere with 5% CO_2_. Passages were made when confluence reached 80–90% by tripsinization with 0.25% trypsin EDTA and gentle mechanical detachment. Cells were routinely evaluated for viability and cell number for passaging (>95% viability, 2 x 10^6^ cells/ 25cm^2^), also viability was evaluated with a resazurin assay at different concentrations of metformin to asses toxicity.

### Clonogenic assay

Cells were seeded into 6 well plates in triplicates at density of 1000 cells/well in 1.5 ml of DMEM high glucose containing 10% FBS. After 24 h, cultures were replaced with fresh culture medium containing 0 mM, 10 mM or 20 mM of metformin in a 37°C humidified atmosphere with 95% air and 5% CO_2_, and grown for 9 days. The culture medium was changed once every 3 days. The cell colonies were fixed with 4% PFA and then stained for 15 min with a solution containing 0.5% crystal violet and 25% methanol, followed by tree rinses with PBS to remove excess dye according to previously published protocols [23]. Colonies consisting of >50 cells were counted under a microscope. Also, all colonies were photographed for later analysis and area measuring with Image J program[24].

### Proliferation assay

Cells (1x10^6^) were seeded in 25 cm^2^ culture flasks. After 24 h medium was removed and replaced with fresh but now containing 1% FBS and incubated for other 24 h (for culture synchronization). After that the cells were harvested by tripsinization and labeled with CFSE 2 uM (Invitrogen, CA, USA) according to the manufacturer instructions. Labeled cells were seeded (5x10^5^) in 6 well plates and left for adherence for 24 h, after this time fresh culture medium containing 0 mM, 10 mM or 20 mM of metformin was added to the cells and cultured for another 24 or 48 hours in a 37°C humidified atmosphere with 5% CO_2_. Proliferation was analyzed using a CANTO II flow cytometer (Becton Dickinson) and Flow Jo 10.0 program considering the cells at time 0 as no proliferation and the cells without metformin as 100% proliferation.

### Cell cycle analysis

Cells (5x10^5^) were seeded in plates. After 24 h the medium was removed and replaced with fresh medium containing 0 mM o 20 mM for 48 h. Cell cycle was analyzed by measuring the amount of propidium iodide (PI)—in ethanol fixed cells. In brief, cells were harvested by tripsinization and fixed with cold 70% ethanol for 24 h. After that were rinsed 3 times with PBS and resuspended in 1 ml of permeabilizing solution (Triton 100x (0.25%), sodium azide (0.01%) and RNAsa A (100μg/μl Sigma-Aldrich) in PBS for 10 min. Rinsed once with PBS, resuspended with 1 ml of PBS with PI (2.5 mg/ml) and incubated for 15 min at 4°C. Cell cycle distribution was analyzed using a CANTO II flow cytometer (Becton Dickinson) at slow flow rate and with doublet discrimination in the Flow Jo 10.0 program and the Cell Cycle analysis feature (Flow Jo, LLC).

### Animal model of wound healing

Thirty-six adult Wistar male rats divided into three groups were used for the animal model of wound healing. The weights of the animals ranged from 250–300 (gr/animal) and were provided by the animal facility at the “Universidad Autónoma de Zacatecas, Campus Siglo XXI”. The Bioethics Committee and Institutional review Board at “Area de Ciencias de la Salud, Universidad Autonoma de Zacatecas” approved the realization of this study, registry number **UAZ201536879.** All animals were healthy and had free access to balanced feeding pellets and water. Housing conditions were as follows: Animals were kept in individual polycarbonate cages, temperature ranged from 22+/-3°C, humidity ranged from 45–70% and light/darkness cycles of 12 hours. All procedures were made according to NOM-062-ZOO-199 (Mexican legislation) for the production, care and use of animals for experimental purposes. Animals were anesthetized in an acrylic chamber with sevofluorane, baseline weight was determined in a certified scale. For the wound, a disposable biopsy punch of 0.8 cm^2^ (HealthLink^®^, Jacksonville, Fl) was used producing a uniform tisular defect in all animals in the dorsal skin. This procedure was performed in aseptic conditions with benzalconium chloride 1% w/v (Antibenzil^®^) to prevent acute infection. Daily clinical inspection of the animals and documentation by referenced photography was performed during 14 days post-lesion. From previous reports, a dose of 300 ug/kg in PBS administrated daily with a stomach probe was used for the metformin treated group. Also a PBS vehicle (with stomach probe) and prednisolone (0.25 milligrams topically) group was included as control. Animals were evaluated at days 0, 3, 7, 10, 12 and 14. Wound closure was evaluated from the photographs were measured from the referenced images using Image J, the ruler in each photograph was used as a standard to set scale for each image. Furthermore, weight (data not shown) and glycemia (Accu-check Performa, Roche, U.S.A.) concentrations were determined and recorded for every animal in each time point as previously reported [25]. At the end of the experiments after the last evaluation (day 14) animals were sacrificed by an overdose of anesthesia (overexposure to anesthetics chamber).

### Analysis of Diabetic foot ulcer patients

The study was approved by “Hospital General: Luz González Cosío” ethics committee at Zacatecas (R-021/2014). In this observational study, data was collected retrospectively from clinical files of patients with diabetes mellitus type II whom suffered a foot ulcer between January 2010 and May 2015. Informed consent was not obtained, only clinical files were reviewed and all information was anonymized and de-identified prior to analysis. Clinical files were reviewed to evaluate the potential effect of metformin as a risk of amputation. Records were divided into two groups, **metformin group** (patients treated with metformin) and the group of **other antidiabetic drugs** (patients treated with others antidiabetic drugs such as sulfonylurea, glybenclamide, insulin or else, except metformin). 95 patients had a diagnosis of diabetic food ulcer and had a complete description of the wound and from these, 71 patients had complete clinical data and information available for analysis. Of these 72, 37 were treated with metformin and the other 34 were treated with other antidiabetics. Clinical characteristics such as age, sex, WBC, time with diagnosis of DM2, time with the ulcerative process, previous amputation, area of ulceration, ischemia, concentration of glucose at the moment of admission to the hospital. Data on ulcer infection agreed with the description given by the treating physician according to the guidelines of the American Society of infectious diseases as the presence of purulent wound drainage or designated as systemic or local inflammatory findings.

### Statistics

Statistical analysis of data was performed using Prism (Graph Pad) and SPSS 22.0 software packages. Normality of data was verified by a D’Angostino-Pearson normality test. For quantitive variables two tailed Student´s t test or Mann-Whitney tests were performed according to normality of data. For proportions and categorical variables a X^2^ or Fisher tests were performed and for the significance of the odds ratio (OR). In the case of more than two groups One-way analysis of variance (ANOVA) with Friedman post test was performed and for animal data, a two way ANOVA was performed to analyze for interaction. A P value <0.05 was considered statistically significant.

## Results

### Metformin reduces cell proliferation without causing toxicity to keratinocytes

**Fig 1:**
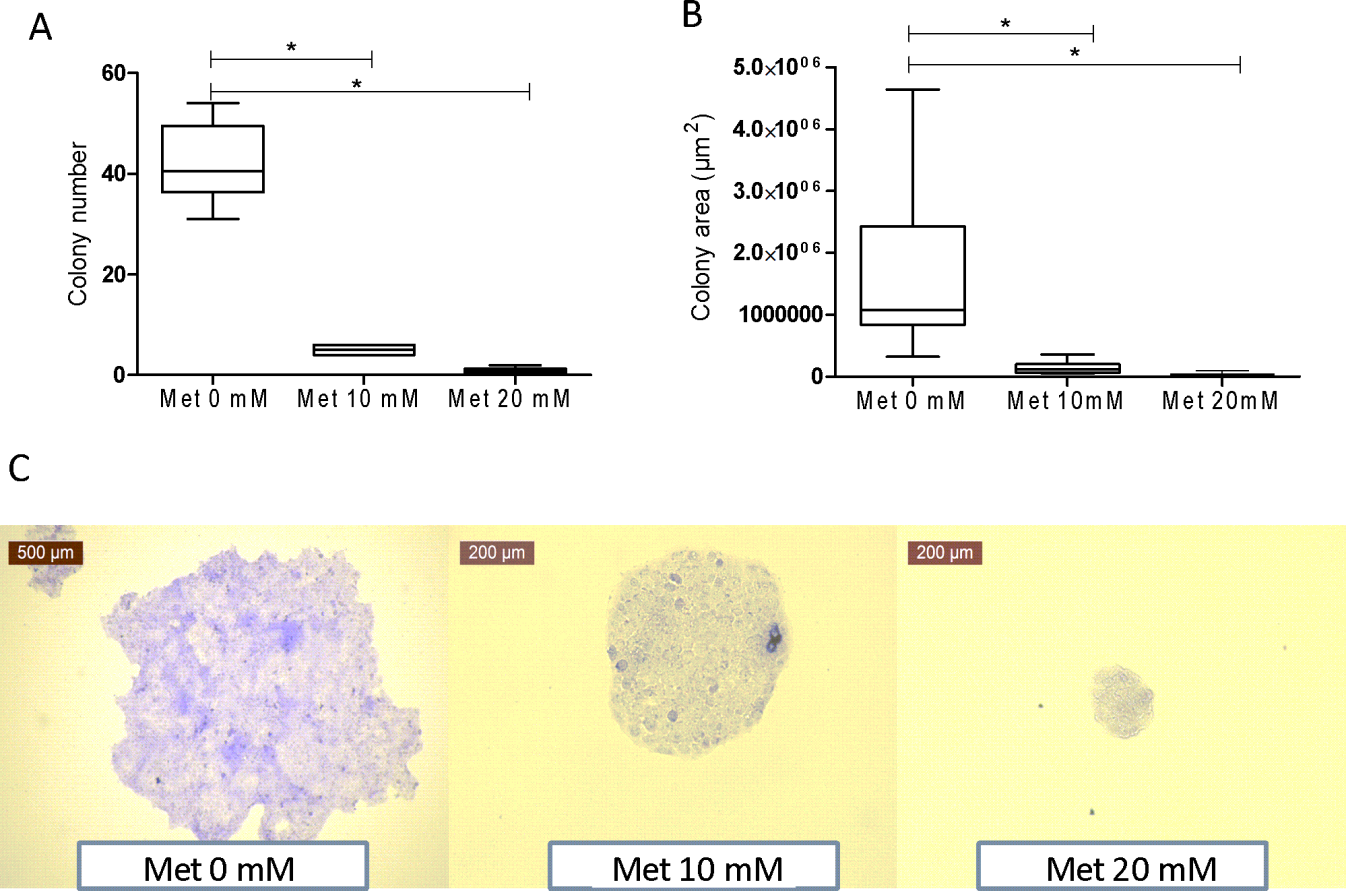
Metformin inhibits HaCaT cell proliferation and colony formation. 1000 cells/well human HaCaT cells were plated onto 6-well and incubated at 37°C with 5% CO_2_. After 24h, the culture medium was replaced with fresh culture medium containing 0.0 mM, 10 mM or 20 mM every 3 days for 9 days. (A) At 9 days the cells colonies were stained and counted as described in the methods section. (B) Colony area was determined using Image J program. (C) Image is representative of the size of colonies observed for each of the treatments. Data are representative of six independent experiments.

Previous reports suggest that metfomin has an important effect on cell proliferation and from there, it has been suggested to be used as an adjuvant therapy in cancer chemotherapy, and even some clinical trials are now undergoing and some results have been reported in this matter trying to demonstrate their effect in the clinical setting [26]. However, the effect of metformin on other cell types that are involved in complications of diabetes such as diabetic foot ulcers, has not been analyzed. Therefore, we performed an experiment to analyze whether metformin could reduce cell proliferation. First, we performed an analysis of toxicity with a resarzurin assay, no significant toxic effects of metfromin were found for a range of concentrations from nanomolar to millimolar concentrations (**Data not shown**). Concentrations of 20 mM have been used as representative of the physiological concentrations of metformin inside the mitochondria [27]. Then we performed an analysis of the effect of metformin on the formation of colonies of >50 cells. A significant reduction in the number of colonies was observed when comparing the control treated vs metformin treated cells as shown in **Fig 1A** (p<0.05), also, a significant effect was observed in both the size (P<0.05 for both treated groups) and morphology of the colonies in the treated and control cells (**Fig 1B and 1C** respectively).

**Fig 2:**
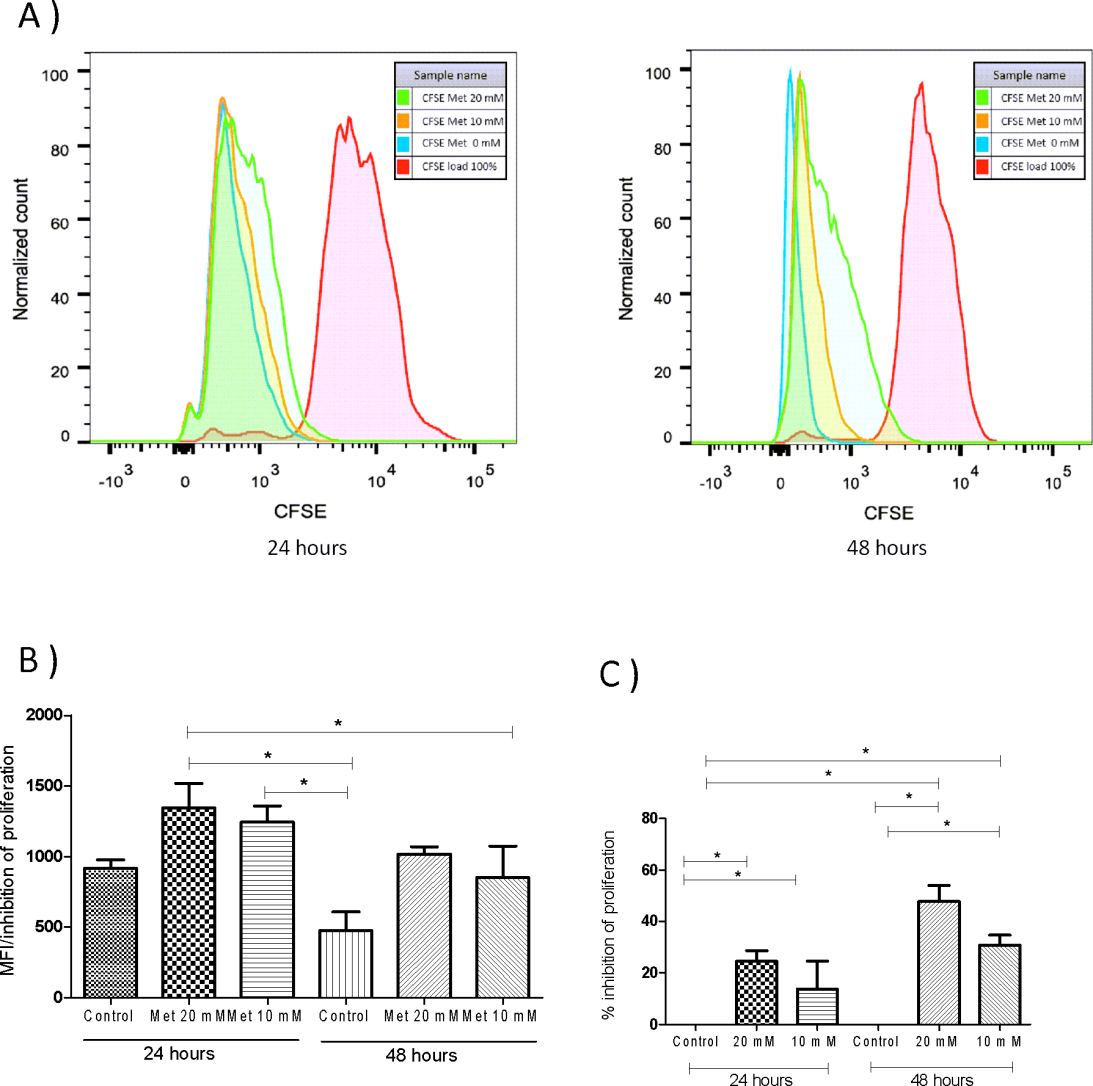
Metformin induced inhibition of HaCaT cells proliferation. Human HaCaT cells were cultured 24 hours before CFSE labeling in FBS 1% to synchronize the culture cell cycle. Labeling was performed with CFSE as described in the methods section. After that the cells were cultured for 24 hours to adherence and then treated with 0 mM (PBS), 10 mM and 20 mM of metformin for 24 or 48 hours. The proliferation in HaCaT cells was assessed by flow cytometry. (A) Histograms show cell proliferation inhibition at 10 mM and 20 mM, the effect is observed after 24 hours of treatment. (B) The graph shows the mean fluorescence intensity of cells labeled with CFSE, it is clear that cells were treated with metformin have a higher intensity of fluorescence due to decreased cell proliferation. (C) Percentage of inhibition of proliferation is shown compared to control treated cells. Inhibition is observed in both the 10mM and 20 mM metformin treated cells and dependence on time is also observed.

To provide further evidence of the effect of metformin on the proliferation of cells, we performed a flow cytometry assay based on the dilution of CFSE. **Fig 2A** shows a normalized histogram of the frequency of cells with different intensities in their fluorescence at 24 and 48 hrs. In each graph the CFSE load (100% stained cells) are the cells that were labeled and immediately fixed with 4% PFA in extreme right of the graph denoting a brighter signal, which fades as cells divide indicating the “dilution” of the dye. It can be observed for the 20 mM metformin treated group that a high proportion of cells retain the fluorescence, indicating that metformin can inhibit cell proliferation at 24 and 48 hrs. Data from multiple experiments was analyzed for the mean fluorescence intensities and statistically significant differences were found for both the MFI (**Fig 2B**, P<0.05) and the percentage of inhibition for the 10 mM and 20mM concentrations of metformin at 24 and 48 hours (**Fig 2C**, P<0.05).

### Metformin treatment alters the cell cycle of keratinocytes and does not induce apoptosis

**Fig 3:**
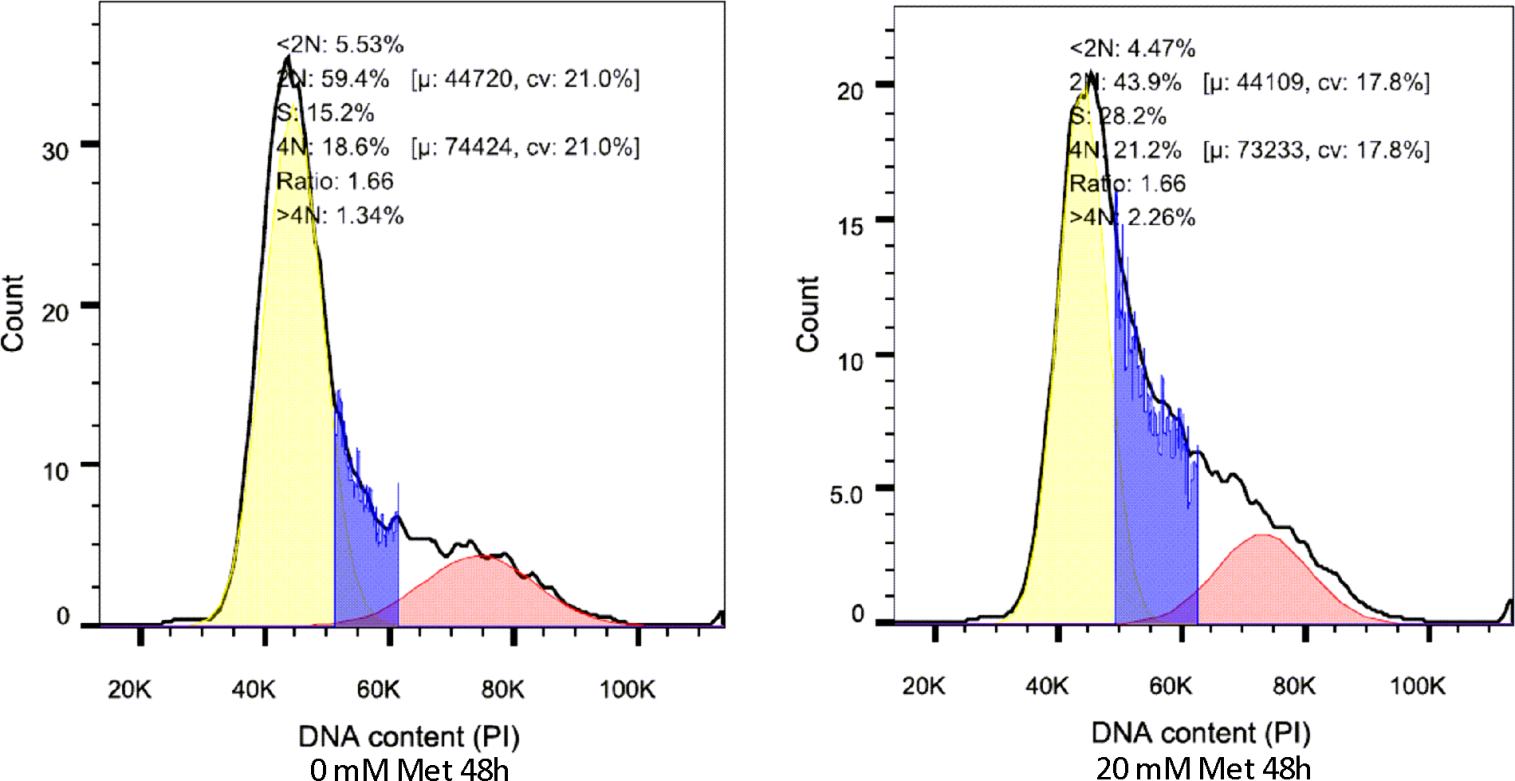
Metformin blocks cell cycle progression. Human HaCaT cells were grown in plate (5 x 105 cells). After 24h the culture medium was replaced with fresh culture medium containing 0 mM or 20 mM metformin for an additional 48h. Cell cycle progression in HaCaT cells was assessed by flow cytometry by analyzing PI fluorescence in slow acquisition and with doublet discrimination, 50000 events were acquired. Histogram showing cell cycle HaCaT with 0 mM and 20 mM of metformin, in the cells treated with 20 mM can be seen an increase in the proportion of cells which are in transition from G0/G1 to S phase. Histograms depicted above are representative of at least three independent experiments, which showed similar results.

Due to the fact that clear alterations in cell proliferation were found for the HaCaT cells in the presence of metformin, we wondered whether metformin was having an effect on the cell cycle. Cell cycle analysis was performed by means of flow cytometry based method using propidium iodide. A representative histogram of the distribution of HacaT cells is depicted in **Fig 3A** untreated and **Fig 3B**, metformin treated 20 mM for 48 hours. A marked increase in the S phase of the cell cycle was observed for the metformin treated cells (from 15.2% in the untreated cells to 30% in the metformin treated condition) providing insights into the mechanism of cell proliferation arrest. Also, as can be observed in the cell cycle analysis histograms, the sub Go/G1 populations is negligible, suggesting that at the concentration and time used of metformin treatment and exposure (respectively), there is no observable induction of apoptosis.

### Metformin reduces the rate of wound healing in an animal model

**Fig 4:**
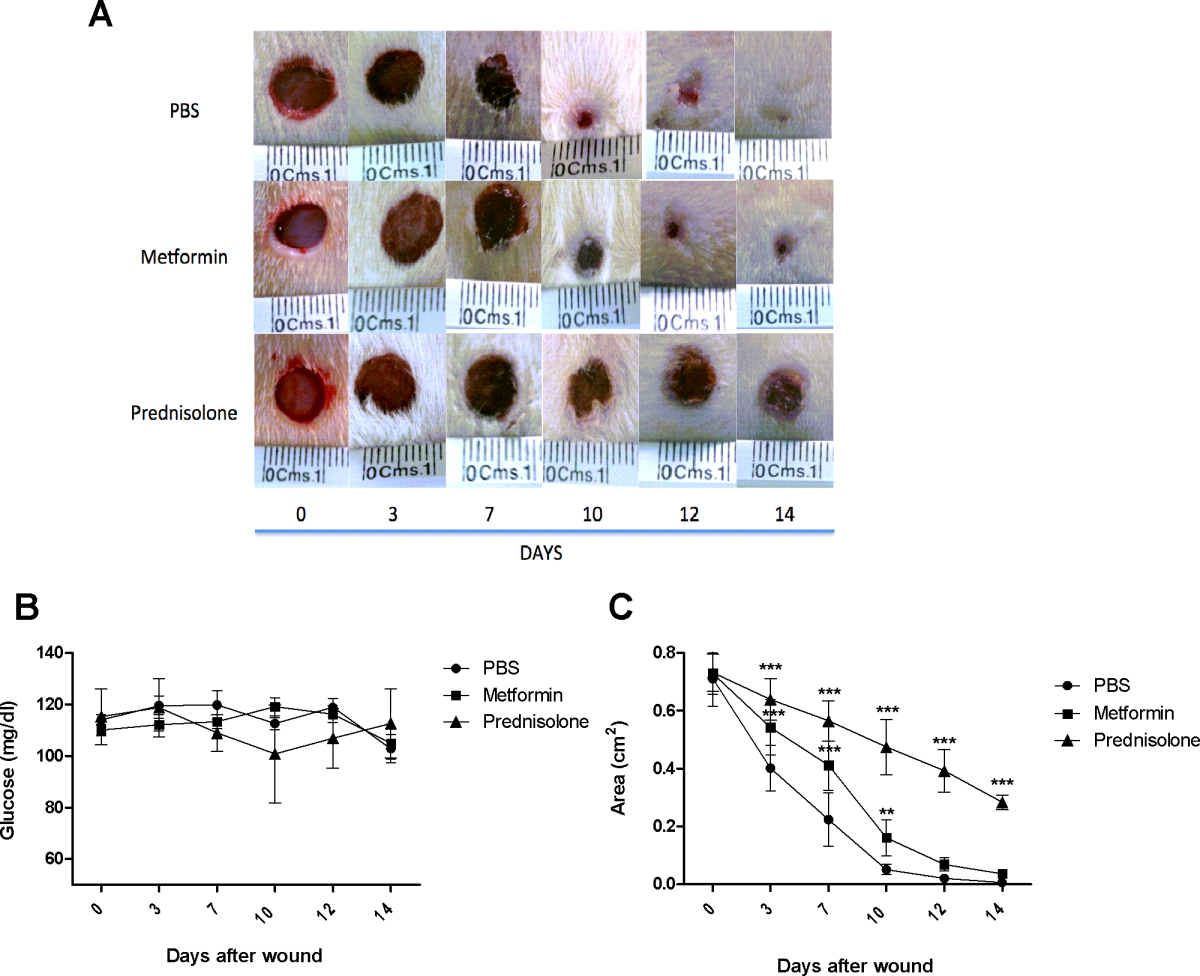
Metformin inhibits wound healing *in vivo*. A rat model of wound healing was used to evaluate the effect of metformin on cell proliferation and wound closure. (A) The panel shows the effect of metformin (300mg/kg) in the healing of a standardized model of wound healing, PBS was used as a control and topical prednisolone (0.25 mg) has been used in this model as control of delayed healing. Representative photographs of each time point are shown. (B) Glucose concentrations were measured daily in the animals by means of a portable glucometer (mg/dl), to monitor the effect of metformin compared to PBS and prednisolone treatment. (C) Ulcer diameters were measured from the referenced images using Image J, the ruler in each photograph was used as a standard to set scale for each image. Groups of animals of N = 12 animals per treatment group were used for the experiments in two independent experiments. Analyses of glucose and area differences were made by means of a Two-way ANOVA. P<0.05 was considered statistically significant.

Once we confirmed in the *in vitro* model that a clear alteration and inhibition of cell proliferation was observed upon metformin treatment and also alterations in the cell cycle, the question remained of whether metformin had an effect in a physiological setting. Therefore, an *in vivo* model of wound healing was used to evaluate the effect of metformin. The rat model has been previously described [25]. Photographic documentation of all animals at the pre-specified time points was performed. **Fig 4A** shows the effect of the metformin treatment at previously reported doses [28] in wound closure. As shown in the panel, an evident reduction in the rate of closure is observed in both the prednisolone and metformin treated groups compared to control (PBS). This is further confirmed in **Fig 4C**, that shows the measured areas of the wounds at different time points. Significant differences in wound areas were identified at 3, 7 and 10 days after the wound was made (P<0.05), suggesting a role for metformin treatment in delaying the wound closure process in which keratinocytes have a major role. The lesions of the animals in the metformin group also had features such as redness and remaining scarring which were not observed in the PBS treated group, thus suggesting that tissue remodeling was also affected by treatment, but this requires further investigation. Given that it has been observed that reduction in glycemia is a factor affecting the rate of proliferation and wound closure in other settings and for this reason the animals in the experiment were closely monitored for glucose control in the experiment. As shown in **Fig 4B**, statistically significant differences in glucose concentration were not found.

### Increased ulcer size in DFU patients is observed in metformin treated patients

**Fig 5:**
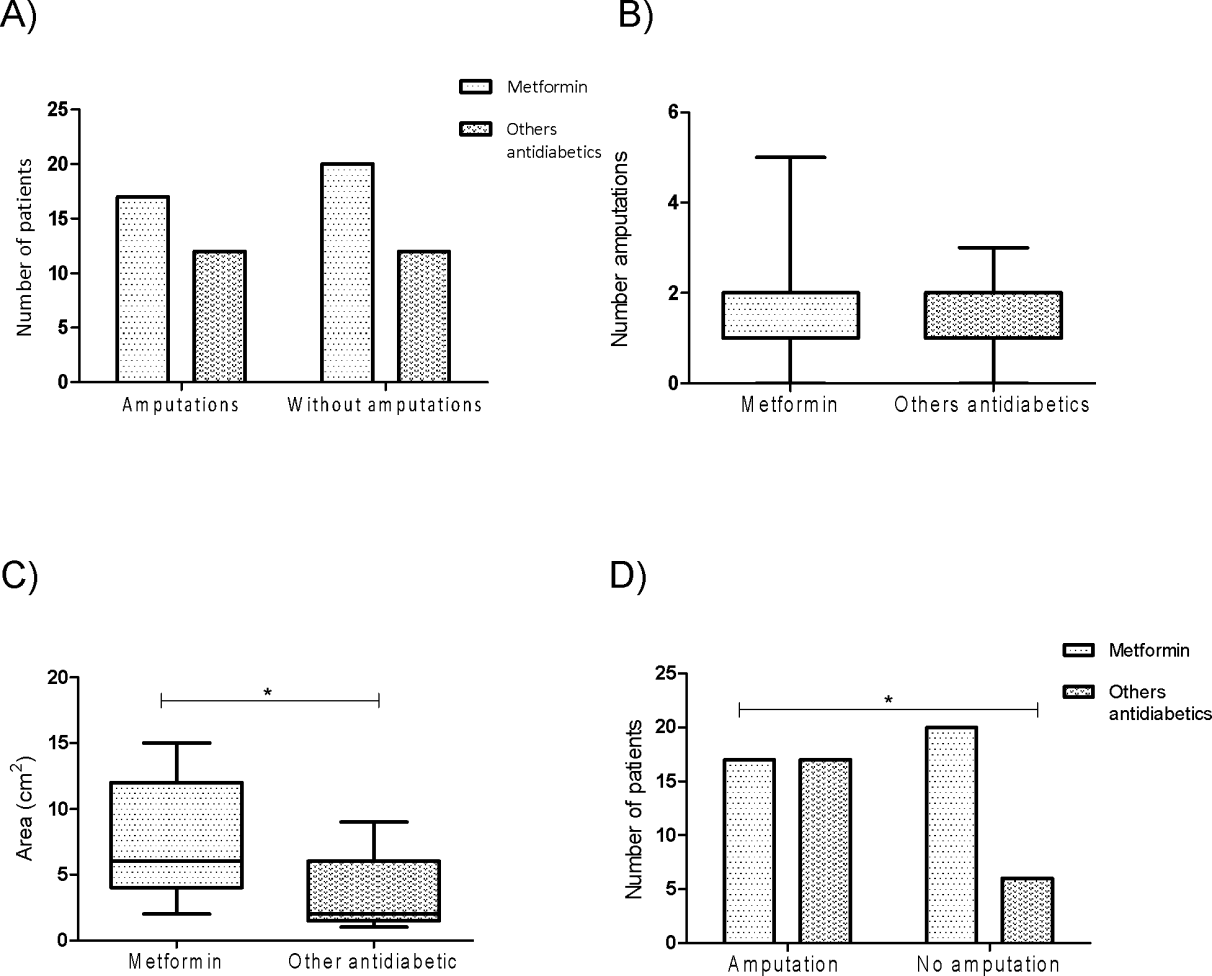
Effect of metformin in history of diabetic foot ulcer. Data shown was taken from the clinical records of patients who had diabetic foot ulcer and stratified by treatment with metformin or others antidiabetic drugs (n = 72). (A) The graph shows patients at the time of the review of the record had already undergone some amputation. (B) The graph shows the number of previous amputations per treatment group. (C) The graph shows the area of ulceration reported by the treating physician, the size of ulcers is greater in the group of patients receiving metformin as part of their treatment for glycemic control (p = 0.029, Mann Whitney test). (D) The graph shows whether patients discharged from the hospital were amputated as a result of the severity of his ulcer, seen as the proportion of subjects amputees is higher in the group of patients treated with other antidiabetic drugs (p = 0.035 Fisher´s exact test). P<0.05 was considered statistically significant.

According to the evidence provided so far, we knew that metformin had an important effect on cell proliferation *in vitro* and *in vivo* and this effect on proliferation reduced the rate at which the wound in the animal model closed. Given that this previous observations might have important clinical repercussions for patients that have chronic wounds such as the diabetic patients with DFU, we analyzed in a group of patients from the “Luz Gonzales Cossio” Hospital that were admitted for chronic ulcers and that had a more complete information in their clinical file, for this analysis seventy three cases were included; from these files, the patients were stratified according to the treatment they were receiving at the time of admission to the hospital. The general characteristics of these patients are described in **Table 1.** Statistically significant differences were found after stratification for the proportion of subjects with infection (P = 0.039). For all the other variables analyzed, no differences were found. From the patients described above, no differences in the proportion of patients with at least one previous amputation were observed (**Fig 5A,** P>0.05), neither difference in the number of previous amputations on those that had at least one previous amputation in both groups (**Fig 5B**, P>0.05). However, when analyzing the area of ulceration between the treatment groups (metformin and other antidiabetics), significant differences were identified for both groups (p<0.05) suggesting that according to our previous data, a reduced proliferation is observed in metformin treated subjects and therefore bigger areas of ulceration are present on average in these patients (**Fig 5C)**.

**Table 1:** Clinical features of patients with foot ulcers.

	Metformin	Other antidiabetic drugs	P value
Gender (male/female)	24/14	20/5	0.175^a^
Age (years), median (IQR)	66 (57–74)	61 (52–70)	0.093^b^
Time with diagnosis of DM2, mean (SD)	13.6 ±6.1	15.9 ± 7.8	0.235^c^
Time with ulcerative process (days) median (IQR)	90 (32–128)	150 (98–248)	0.133 ^b^
Time of hospitalization (days), median (IQR)	5 (4–7)	4 (3–7)	0.350 ^b^
BMI (kg/m^2^), median (IQR)	25.6 (23.5–29.3)	25.4 (21.9–26.9)	0.419^b^
Ischemia (yes/no)	22/7	12/4	1.000^a^
Infection (yes/no)	25/10	20/1	0.039^a^
Glucose (mg/dL), median (IQR)	216.3 (144.2–283.0)	203.5 (131.0–369.0)	0.964^b^
Hemoglobin (g/dL), median (IQR)	11.5 (9.9–12.4)	10.0 (9.7–11.7)	0.320 ^b^
WBC	12.5 (9.0–13.4)	11.6 (9.7–12.6)	0.657 ^b^

^a^ Subject proportions calculated by Fisher 's exact test

^b^Comparing groups calculated with Mann Whitney test

^c^ Comparing groups calculated with t test.

Differences in the number of patients in the variables ischemia and infection are due to the fact that no description of such variable was available for several patients.

### Metformin is associated with positive outcomes in DFU patients

According to these observations, and given that chronicity of ulcerations is a risk factor for amputation (due to the increased chances of infection and vascular complications), we analyzed whether patients at discharge from the hospital had been subjected to amputation. Unexpectedly, as shown in **Fig 5D**, when analyzing for differences in the proportion of subjects with amputation, a significantly higher proportion of subjects without amputation were on metformin treatment, suggesting that metformin treatment is protective of amputation (OR = 0.30 CI = 0.1–0.9 P<0.05).

## Discussion

Metformin is an important therapeutic agent in diabetic patients with poor glycemic control. The widespread use of this drug in diabetes treatment has been linked to reduced incidence of several types of cancer for their reported effect on cancer cell proliferation [16, 26, 28, 29].

Under the premise that the same phenomenon might occur in non-cancerous cells implicated in wound closure, aggravating the ulcers of diabetic patients. We wondered if this was the case and if metformin inhibited cell proliferation and had effects on wound closure. Metformin is one of the most widely used drugs among diabetics and in recent clinical trials it has shown an improvement in the vascular function and reduction of vascular complications and therefore of cardiovascular events beside it´s glucose lowering effects [30]. This is to our knowledge the first report regarding the role of metformin on keratinocyte proliferation in non-transformed or tumor cell lines, in the role that it might have *in vivo* and in the clinical setting. We provide evidence that metformin can reduce cell proliferation in keratinocytes by different methodologies and the data also suggest that the mechanism is not mediated by direct toxicity to the cells. Several reports indicate that metformin might induce apoptosis [31], but it is not the case in HaCaT keratinocytes as shown for the analysis of cell cycle in which the subG1/G0 populations are negligible in both the metformin treated and control samples; this suggests that at the time points and concentrations used of metformin in this study, no apoptosis was induced.

A marked effect on proliferation was observed in the clonogenic assay and the CFSE assay. The mechanism mediating this inhibitory effect observed on cell proliferation might be associated with the altered cell metabolism that is induced by metformin inhibiting the electron transport chain, particularly the complex I and complex III and IV which has been suggested to have significant effects on the activity of these multimeric complexes [32], suggesting that the effects of metfromin are ultimately mediated by ATP synthesis in an indirect manner, although, other mechanisms have been suggested such as inhibition of AMPK and mTOR [33, 34]. The latter being the most likely as has been recently reported in HaCaT keratinocytes [35]. The reduced proliferation of HaCaT cells and changes in the cell cycle were clearly associated with an S phase of the cell cycle and it has been already reported that inhibition in cell cycle by metformin might be associated with a diminished activation or production of Cyclin D/CDK4 (the main controller of S to G2 cell cycle phases) [36, 37] or Cyclin A/CDK2 that has been reported to be active during the S phase and associated with AMPK, a target regulated by metformin [38].

The observed effect on proliferation is not exclusive of HaCaT as other cell types that play important roles in wound healing of diabetic foot ulcers are also affected. It is known that immune cells such as CD8+ [39] and endothelial cells are also affected by metformin, in a similar manner [40], therefore making it even more plausible to be involved in the deregulated vascularization and wound healing observed during metformin treatment.

Our results are consistent in the *in vitro* and in the *in vivo* experiments showing that metformin treatment delays the wound healing process and that this process is independent of the blood glucose levels that have important effects on cell proliferation [41]. Several important features were observed in the metformin treated groups related to the wounds in these animals, particularly, the lesions in the treated animals were less remodeled than those in the control group. This suggests that important mediators of tissue remodeling such as MMP´s, inflammatory cells and the inflammatory process and others mediators might be affected in this process [42], furthermore, given that metformin treatment is known to diminish activation of NF-kB [40], it is very likely that inflammation is reduced due to treatment and with it all these mechanisms as well. The effects of metformin in a diabetic animal model of wound healing needs also to be further explored given that recent reports suggest that metformin treatment (in a sustained release 3D matrix) improves the healing capacity [43] of the wounded animals, although given the differences in administration, it is likely that concentrations of metformin are higher in these experiments and it has been reported that different effects in the clinical setting arise from different doses of the drug, thus probably affecting different pathways one mediated by complex 1 inhibition (leading to sensitization to insulin) and the other leading to AMPK activation at different concentrations and also the underlying differences of the model [44]. The immune cells that infiltrate these lesions as well as the molecules (cytokines, chemokines, etc) that participate in these processes need to be further elucidated.

Once we knew that metformin treatment had an important effect *in vitro* and knowing the plausible mechanism of such inhibition we wondered whether metformin treatment might have significant clinical effects in diabetic patients and their diabetic foot ulcers. We found an increased ulcer diameter on the treated patients, these suggested that patients with metformin treatment had a reduced proliferation in their keratinocytes and therefore, an increased diameter in their ulcers, however, these observations need to be confirmed in a prospective cohort to support their causal association. According to our observations, the reduced proliferation of keratinocytes increases the time to heal of these patients and increasing the risk of infections and therefore of amputation. It has been reported that severity of infections accompanied of systemic signs of infection such as fever or edema are associated with increased risk of amputation [13]. For this reason we performed a risk analysis in a contingency table, under the premise that DFU patients that were on metformin treatment would be more prone to be amputated at the time of discharge from the hospital. Surprisingly, a protective effect of metformin was observed according to which, patients in metformin treatment were less prone to amputation (as observed in the (<1) OR. This protective effect might be associated with the role that has been reported of metformin in reducing inflammation [45–47] and this is further supported by the fact that inflammatory mediators are good predictors of amputation and further supporting the idea that the control of proinflammatory mediators such as C reactive protein might reduce the incidence and frequency of amputations in diabetics [13]. Another way by which metformin might be associated to this protective effect is through induction of autophagy, as has been already described in latent TB infection by increasing autophagy and other microbicidal mechanisms in an mTOR dependent mechanism [48] although it has been only reported in intracellular infections.

Further research is needed to clarify whether metformin treatment or other factors are associated with the findings we report in the present work. Other factors such as metformin treatment adherence, metabolic control, time of diabetic diagnosis, age, and others might be obscuring these findings.

Several limitations of the study can be mentioned such as sample size. Also, the study design that would better inform of such causal relationship of metformin treatment and delayed wound closure in the clinical setting is a prospective study, however, the associations found in the study are supported by strictly controlled, *in vitro* and *in vivo* experiments, guarantying further research in this topic to confirm or reject the observed effects of metformin and their clinical significance.

Based on previously reported observations, metformin treatment probably has important effects on the AMPK/electron transport chain, altering the ratio of ADP/ATP. These alterations as previously reported in numerous *in vitro* models, and could be responsible for the reduced proliferation of keratinocytes and altered cell cycle and in turn would also be responsible for the reduced proliferation and delayed wound healing as well as tissue remodeling contributing to increased wound size and increasing the time for complete healing of the lesions. Similar events as the ones described earlier would be observed in diabetics with DFU, and their ulcers with important differences in reducing the incidence of amputations, probably due to a boosting in the anti-inflammatory and antimicrobial effects of metformin.

## Supporting Information

S1 Data Raw data. The file contains the unprocessed data from the experiments in a xls format.
(XLSX)
