# Correction: Metformin Induces Cell Cycle Arrest, Reduced Proliferation, Wound Healing Impairment *In Vivo* and Is Associated to Clinical Outcomes in Diabetic Foot Ulcer Patients

**DOI:** 10.1371/journal.pone.0159468

**Published:** 2016-07-12

**Authors:** Fatima Ochoa-Gonzalez, Alberto R. Cervantes-Villagrana, Julio C. Fernandez-Ruiz, Hilda S. Nava-Ramirez, Adriana C. Hernandez-Correa, Jose A. Enciso-Moreno, Julio E. Castañeda-Delgado

There is an error in affiliation for author Julio E. Castañeda-Delgado. The correct affiliation should be: CONACYT—Unidad de Investigación Biomédica de Zacatecas, Instituto Mexicano del Seguro Social (IMSS), Zacatecas, Zac., México.
